# Distribution of tract deficits in schizophrenia

**DOI:** 10.1186/1471-244X-14-99

**Published:** 2014-04-02

**Authors:** Ian Ellison-Wright, Pradeep J Nathan, Edward T Bullmore, Rashid Zaman, Robert B Dudas, Mark Agius, Emilio Fernandez-Egea, Ulrich Müller, Chris M Dodds, Natalie J Forde, Cathy Scanlon, Alexander Leemans, Colm McDonald, Dara M Cannon

**Affiliations:** 1 Department of Psychiatry, Brain Mapping Unit, University of Cambridge, Herchel Smith Building for Brain and Mind Sciences, Robinson Way, Cambridge CB2 0SZ, UK; 2 Avon and Wiltshire Mental Health Partnership NHS Trust, Heathwood, Fountain Way, Salisbury SP2 7FD, UK; 3 GlaxoSmithKline, Clinical Unit Cambridge (CUC), Addenbrooke’s Centre for Clinical Investigation (ACCI), Addenbrooke’s Hospital, Hills Road, PO Box 128, Cambridge CB2 0GG, UK; 4 School of Psychology and Psychiatry, Monash University, Building 17, Clayton Campus, Wellington Road, Clayton, VIC 3800, Australia; 5 Department of Psychiatry, University of Cambridge School of Clinical Medicine, Cambridge Biomedical Campus, Box 189, Cambridge CB2 2QQ, UK; 6 South Essex Partnership University NHS Foundation Trust (SEPT), The Lodge, The Chase, Wickford, Essex SS11 7XX, United Kingdom; 7 Cambridgeshire and Peterborough NHS Foundation Trust (CPFT) Elizabeth House, Fulbourn Hospital, Fulbourn, Cambridge CB21 5EF, UK; 8 Behavioural Clinical Neuroscience Institute (BCNI), University of Cambridge School of Clinical Medicine, Cambridge Biomedical Campus, Box 189, Cambridge CB2 2QQ, UK; 9 Clinical Neuroimaging Laboratory, Departments of Anatomy & Psychiatry, College of Medicine, Nursing and Health Sciences, 202 Comerford Suite, Clinical Sciences Institute, National University of Ireland, Galway, Republic of Ireland; 10 Image Sciences Institute, University Medical Center Utrecht, Q.S.459, P.O. Box 85500, 3508 GA Utrecht, The Netherlands; 11 New Medicines, UCB Pharma, Chemin du Foriest B-1420, Braine-l'Alleud, Belgium

**Keywords:** Schizophrenia, Diffusion tensor imaging, Tract based spatial statistics, Voxel based morphometry, Gray matter, White matter

## Abstract

**Background:**

Gray and white matter brain changes have been found in schizophrenia but the anatomical organizing process underlying these changes remains unknown. We aimed to identify gray and white matter volumetric changes in a group of patients with schizophrenia and to quantify the distribution of white matter tract changes using a novel approach which applied three complementary analyses to diffusion imaging data.

**Methods:**

21 patients with schizophrenia and 21 matched control subjects underwent brain magnetic resonance imaging. Gray and white matter volume differences were investigated using Voxel-based Morphometry (VBM). White matter diffusion changes were located using Tract Based Spatial Statistics (TBSS) and quantified within a standard atlas. Tracts where significant regional differences were located were examined using fiber tractography.

**Results:**

No significant differences in gray or white matter volumetry were found between the two groups. Using TBSS the schizophrenia group showed significantly lower fractional anisotropy (FA) compared to the controls in regions (false discovery rate <0.05) including the genu, body and splenium of the corpus callosum and the left anterior limb of the internal capsule (ALIC). Using fiber tractography, FA was significantly lower in schizophrenia in the corpus callosum genu (*p* = 0.003).

**Conclusions:**

In schizophrenia, white matter diffusion deficits are prominent in medial frontal regions. These changes are consistent with the results of previous studies which have detected white matter changes in these areas. The pathology of schizophrenia may preferentially affect the prefrontal-thalamic white matter circuits traversing these regions.

## Background

Schizophrenia is a multi-symptom disorder characterised by abnormalities in various domains of mental functioning including hearing (auditory hallucinations), belief (delusions), logical thinking (thought disorder), motivation and emotion [1]. However, despite the variety of symptoms and evidence of brain structural changes [2-6], identification of the neuropathology of schizophrenia has proved elusive [7].

There is increasing interest in elucidating brain connectivity (connectomics), including white matter architecture, and the application of this knowledge to psychiatric disorders [8,9]. The investigation of white matter tract changes in schizophrenia may be fruitful for several reasons. Firstly the tracts comprise axonal bundles inter-connecting gray matter regions. Various theories of schizophrenia propose that schizophrenia is a disorder of connectivity [10,11], specifically of anatomical connectivity [12-14]. Therefore the white matter tracts may constitute the location of pathology. Identification of the most severely affected tracts would provide targets for further neuropathological study. Secondly, there is controversy regarding whether white matter changes are global and microscopic [15-18] or regional and macroscopic [19]. If the distribution of tract changes is uniform then this would provide support the former theory. Thirdly, gray matter changes are now well-characterised in schizophrenia [5,6] and if there are microstructural aberrations in white matter tracts then it is important to discover whether they correspond anatomically with the regions of gray matter change [20]. Fourthly, maximum white matter tract changes may, by analogy with other neuropsychiatric disorders [21,22], be associated with the anatomical (gray matter) regions of primary pathology. Finally, identification of a characteristic spatial pattern of white matter tract differences could provide a key to assist classification and diagnosis using neuro-imaging [23].

The quantification of white matter microstructural organisation can now be achieved more directly using Diffusion Tensor Imaging (DTI) [24-26]. This can quantify a structural measure called fractional anisotropy (FA), which is increased in areas where axons are spatially aligned (and water molecules can more easily diffuse along the axis of the white matter fibers) compared with areas where axons are not aligned or regions with a higher density of cell bodies (e.g. gray matter).

Diffusion Tensor Imaging studies have identified reduced fractional anisotropy in a number of regions and tracts in schizophrenia [2,5,8,27]. These include the corpus callosum [2,5,28-38], Anterior Limb of the Internal Capsule regions [5,28,33,35,38-41], corona radiata [28,35], cingulum bundle [2,5,33,35,38,42,43], inferior fronto-occipital fasciculus (IFOF) [5,28,33,44], superior fronto-occipital fasciculus (SFOF) [28,33], uncinate fasciculus [2,33,38,42,43], fornix [5,28,33,43], superior longitudinal fasciculus (SLF) [2,28,34,35,38], inferior longitudinal fasciculus (ILF) [5,28,31,34,44], optic radiations [28], hippocampus and cerebellum [2].

Our methodological approach was novel in applying triple analyses to diffusion tensor images (DTI) from patients with schizophrenia and control subjects. Our objective was to combine a brain-wide voxel-based approach (TBSS) (which is highly automated) to guide subsequent tract-based analysis (which is more labour-intensive but has the potential for greater sensitivity in detecting changes because of greater anatomical specificity) as well as an atlas-based analysis (permitting quantification of the magnitude of regional changes and providing data for future meta-analyses).

We postulated that gray matter reduction would be present in schizophrenia in regions identified by meta-analysis of previous studies [3]. These studies have implicated a frontal-thalamic circuit in the pathology of schizophrenia [13,40,45,46]. We postulated that patients with schizophrenia would show FA reductions in the Anterior Limb of the Internal Capsule (ALIC) (part of the frontal-striatal-thalamic circuit) as well as reductions in the white matter tracts passing through the ALIC.

## Methods

### Participants

Patients were recruited from Cambridgeshire and Peterborough and South Essex Partnership University NHS Foundation Trusts and control participants from the GlaxoSmithKline healthy volunteer panel. We aimed to match the groups for age, gender, premorbid IQ and handedness (see Demographics in Results section).

Power analysis, based on fractional anisotropy values detected in a previous large study [28], indicated that a sample of 23 subjects in each group would provide statistical power over 80% (testing for reduced FA in the corpus callosum in schizophrenia; one-tailed test, alpha level 5%). From the original sample, 21 patients and 21 controls completed the imaging protocol (reducing power to 70%).

The 21 patients met DSM-IV diagnoses of chronic schizophrenia (n = 19) or schizoaffective disorder (n = 2) using the Mini International Neuropsychiatric Interview and had no other Axis 1 disorders. They were clinically stable for three months, and prescribed unaltered atypical antipsychotics for two months. Participants were excluded if they were prescribed antidepressants, anxiolytics, antiepileptics, anticonvulsants, hypnotics, sedatives or non-prescription medications including vitamins, herbal and dietary supplements. Patients were free of drugs of abuse (except nicotine).

There were 17 males and 4 females, mean age was 34.2 (SD 10.9), with mean pre-morbid IQ (National Adult Reading Test) of 110 (SD 7.0). Patients were moderately/severely symptomatic with a mean total PANSS score of 50 ± 13. Their mean Chlorpromazine equivalent dose was 247 mg.

The 21 control participants had no history of any physical or psychiatric disorders as assessed using a semi-structured physical examination and MINI assessment. They were free of medication or drugs of abuse. There were 14 males and 7 females, mean age was 31.5 (SD 9.1), and mean IQ 112 (SD 5.0).

The study protocol was approved by the Cambridgeshire 3 Research Ethics Committee. Subjects were assessed by their clinicians as having capacity to give written informed consent which was obtained from all participants.

### MRI protocol

Whole-brain structural MRI data were acquired using a GE Signa Twinspeed HDxt system (3 Tesla, 3D sagittal BRAVO fast sequence, flip angle 9°, TE 3.0 ms, TR 7.9 ms, TI 900 ms, ASSET × 2 FOV 256 × 256 mm, matrix 256 × 256 × 252, NEX = 0.5, voxel dimensions 1×1×1 mm, acquisition time = 5:05 min).

Diffusion data was acquired using a 2D axial diffusion Spin Echo EPI sequence (with ASSET). 30 diffusion gradient directions were used with b = 1,000 s/mm2 and one b = 0 s/mm2 reference image (TE 77.2 ms, TR 15,000 ms, FOV 320 × 320 mm2, matrix 128 × 128, in-plane voxel dimensions 2.5 × 2.5 mm2, slice thickness 2.5 mm, acquisition time 8.15mins) [47].

### Image processing

T1-weighted structural MR images were corrected for non-uniform bias using N3 [48], brain extraction was performed using the FSL BET [49], and registered to the FA diffusion images using the FSL FLIRT [50]. Inspection of T1 data revealed 7 subjects (5 patients and 2 controls) had motion artefacts. These were excluded from the VBM analyses.

Diffusion-weighted MR data quality was determined [51]. Images were processed using *ExploreDTI* Version 4.8.1 (http://www.ExploreDTI.com) and corrected for subject motion/eddy current induced distortions [52]. The RESTORE approach [53] was used to estimate the diffusion tensor.

#### Voxel-based morphometry (VBM) of Gray and White matter

The VBM5.1 toolbox (http://dbm.neuro.uni-jena.de), a SPM5 extension, was used to segment T1 images into gray matter (GM), white matter (WM) and cerebrospinal fluid (CSF) tissue maps and to normalise these maps to standard Montreal Neurological Institute (MNI) space.

Images were co-registered to MNI space using a 12-parameter affine transformation and segmented into gray matter, white matter and CSF without the use of prior tissue information; a hidden markov random field (HMRF = 0.3) was incorporated to improve segmentation. Further default VBM5.1 parameters were used (bias regularisation = 0.0001, bias FWHM cut off = 70 mm, sampling distance = 3). Non-linear spatial transformation default parameters were used to normalise each subject to GM and WM MNI tissue templates (warping regularisation = 1 and warp frequency cut-off = 25). Voxel intensity values were modulated by the Jacobian determinant. Modulation was calculated on the non-linear transformation to remove the effects of global brain size differences on local brain structures. Modulated GM/WM images were smoothed with an isotropic 8-mm FWHM Gaussian kernel. Independent 2-sample t-tests were used to test for voxelwise group differences. A False Discovery Rate (FDR) of p ≤ 0.05 was applied to correct for multiple comparisons [54].

#### Voxel-based analysis of fractional anisotropy (FA)

Corrected FA images were analysed using Tract Based Spatial Statistics (TBSS) [55] in FSL. FA images underwent non-linear registration to target FMRIB58_FA standard space image. A study-specific skeleton was generated. For the binary skeleton mask, an optimal FA threshold of 0.3 was chosen following visual inspection.

A general linear model was used to test FA differences between the schizophrenia and control groups, with age as a covariate. Threshold-free cluster enhancement (TFCE) was applied to a permutation analysis with 10,000 random permutations to correct for multiple comparisons [55].

#### Regional distribution of fractional anisotropy (FA) differences

Regional FA differences were localized using the John Hopkins University white matter atlas (ICBM-DTI-81) which parcellates white matter into 50 core regions [56]. The FA values within the study-specific skeleton mask within each region were averaged and percentage differences between the groups were calculated.

#### Tractography-based analysis of fractional anisotropy (FA) differences

Deterministic tensor-based tractography was used to detect tracts of interest. Tracts were identified using ExploreDTI 4.8.1 with a seed point resolution of 3×3×3 mm, a step size of 1 mm, fibre length range equal to 50-500 mm, and an angle threshold of 45°. FA thresholds were optimized by visual determination of the optimal balance between sensitivity and selectivity for the anatomy of each tract of interest (genu, splenium and body of the corpus callosum 0.38, ALIC and cingulum bundle 0.32). Thresholds were chosen well below the averages for the tracts in question so do not affect the reconstruction of the tract of interest but eliminate spurious tracts.

#### Tract definition and segmentation

From the regions with the highest T-statistics, tracts were selected for further investigation: the genu of the corpus callosum, right and left ALIC, right and left cingulum bundle, right and left Superior Longitudinal Fasciculus (SLF), body and splenium of the corpus callosum. Tracts were defined using the FA-independent and anatomically detailed structural T1-weighted MR images. Tract volume was controlled for by covarying tract median FA for tract volume and normalized for median segment length to account for variance in the segment length across subjects [57].

The tracts were defined anatomically as detailed below using ‘inclusion gates’. For each tract in each individual subject, two anatomical structures were defined in the brain (‘gates’) based on defined anatomical landmarks. A tract consisted of all reconstructed paths passing through both anatomically defined gates.

#### Corpus callosum

Segments of the CC were defined mid-sagittally. Borders were located one-voxel external to the CC white matter inferior/anterior, inferior/posterior and superior/inferior for the genu, splenium and body of corpus callosum (BCC) respectively. The genu’s posterior border was defined as one slice posterior to the posterior flexure of the genu, this was also the BCC’s anterior border. Its posterior border was located 25 mm posterior to this. Genu tracts were cut 2 slices anterior to the anterior of the genu in the mid sagittal plane. Splenium fibers were cut at their most lateral extent. The BCC lateral projections were cut sagittally 10 mm from the mid-sagittal line.

#### Anterior Limb of the Internal Capsule (ALIC)

This was defined by a modified previous protocol [58]. The anterior limit was the anterior-most slice of the lateral ventricle and posteriorly the anterior commissure. The anterior gate was bordered by gray matter and medially by the lateral ventricle. The posterior gate was bordered superomedially by the lateral ventricle and inferolaterally by the pallidum and putamen.

#### Superior Longitudinal Fasciculus (SLF)

This was defined by a modified previous protocol [58]. The anterior coronal limit was the first slice anterior to the superior portion of the fornix and posterior limit as the inferior-most slice of the CC splenium.

#### Cingulum bundle

The anterior limit was the posterior flexure of the CC genu and posterior limit the anterior CC splenium. The anterior gate was a frontal lobe wedge, using the mid-sagittal plane medially and axial plane bisecting the genu inferiorly from the mid-sagittal plane to the lateral ventricle. The posterior gate was a wedge of parietal lobe, using the mid-sagittal plane as a medial border and the axial plane bisecting the splenium as an inferior border from the mid-sagittal plane to the lateral ventricle.

#### Reliability

A single rater (NF) defined all tracts. Her reliability and consistency in defining these anatomically was examined by blinding the rater to the images and re-defining the same tracts twice - this achieved an acceptable level of reliability of greater than 86% in defining tracts (ICC = 0.86-0.99).

One subject was excluded in the case of the left ALIC, right and left cingulum bundle, for failing to show adequate tracts. Six further subjects were removed from the left cingulum bundle analysis for failing to show adequate tracts.

#### Statistical analyses

Fractional anisotropy is non-parametrically distributed across tracts so the median FA was employed for each tract in each person rather than mean.

The Shapiro-Wilks test and Levene’s test were used to determine normality of distribution and homogeneity, respectively, for median FA of the tracts and age of subjects. An analysis of co-variance (ANCOVA) was used to compare diagnostic groups, covarying for age and tract volume. Independent samples *t*-test was used to test for differences in tract FA between diagnostic groups. Mann Whitney U was employed to compare age between groups. A chi-square test was used to assess the gender proportions between diagnostic groups.

Our primary analyses were of: (i) the voxel-based analysis of gray matter between the two groups, (ii) the voxel-based analysis of fractional anisotropy and (iii) the tract FA in the right and left ALIC. Our secondary analyses were of the FA values in the corpus callosum, splenium, SLF and cingulum bundle. Our tertiary analyses were of the regional distribution of FA along the TBSS skeleton in each region of the White Matter Atlas. As the study employed multiple analysis methods and many brain volume measurements are correlated, we did not correct for multiple testing (other than the False Discovery Rate used in the voxel-based analyses) and the probability statistics need to be interpreted in this context.

Statistical analyses were carried out using PASW statistics.

## Results

### Demographics

The groups did not differ significantly in gender proportion (p = 0.30), IQ (p = 0.25), or mean age (p = 0.39) (age was non-normally distributed, W = 0.94, p = 0.02). All subjects were right-handed (Edinburgh Handedness Inventory).

### Voxel Based Morphometry (VBM) of gray and white matter

No significant group differences were found between the schizophrenia and control groups.

### Voxel-based analysis of Fractional Anisotropy (FA)

**Figure 1:**
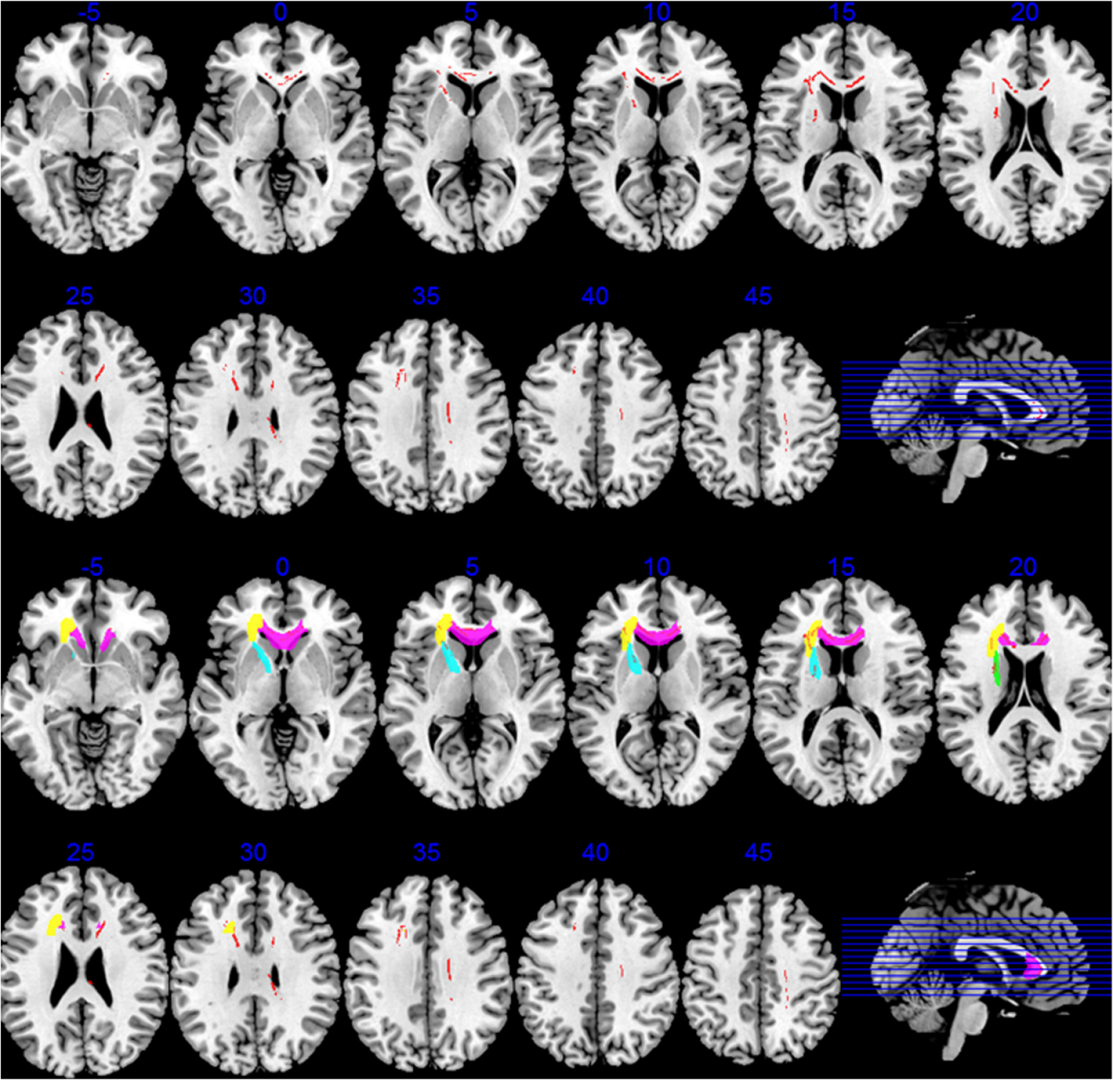
**Regions where the schizophrenia group showed significantly lower fractional anisotropy (FA) compared to healthy controls. Upper two rows:** The regions of reduction detected by Tract-based spatial statistics (TBSS) are shown in red (false discovery rate p < 0.05) superimposed on illustrative axial slices (Talairach level shown above each slice; left brain on left side of image). Reductions are particularly concentrated in medial frontal sectors. Lower two rows: For comparison with the white matter atlas parcellation findings, the white matter regions corresponding to the following are shown in colour (superimposed on the TBSS reductions in red): genu of corpus callosum (atlas region 3; pink), left anterior corona radiata (atlas region 23, yellow), left anterior limb of the internal capsule (atlas region 17, blue) and left superior fronto-occipital fasciculus/anterior limb of the internal capsule (atlas region 43, green).

The schizophrenia group showed significantly lower FA compared to the healthy controls in several regions (Table 1). FA was reduced in schizophrenia (p < 0.05, FDR) in: (i) the genu, body and splenium of the corpus callosum, (ii) the left ALIC, (iii) the left superior fronto-occipital fasciculus/ALIC and (iv) the corona radiata (left anterior and superior, right superior and posterior) (Figure 1).

**Table 1:** Regions in which the schizophrenia group showed significantly lower fractional anisotropy (FA) compared controls

**Region as defined by the white matter atlas**	**T-statistic maximum**	**Talairach coordinate**		
		**x**	**y**	**z**
Genu of corpus callosum	4.4	-13	28	14
Anterior limb of internal capsule (ALIC) left	3.8	-21	1	17
Anterior corona radiata left	3.6	-25	24	15
Body of corpus callosum	3.5	15	-21	31
Superior corona radiata left	2.8	-22	-1	19
Superior fronto-occipital fasciculus/Anterior limb of internal capsule (ALIC) left	2.8	-21	2	19
Superior corona radiata right	2.7	20	-16	39
Posterior corona radiata right	2.5	18	-32	34
Genu of corpus callosum	2.4	13	28	-4
Splenium of corpus callosum	2.3	17	-33	32
Anterior limb of internal capsule (ALIC) left	2.0	-21	-5	16

Regions analysed using Tract-based spatial statistics (TBSS) with false discovery rate p < 0.05. The Talairach co-ordinate is shown for the maximum T statistic within each region.

The schizophrenia group did not show any regions of significantly greater FA than controls.

### Regional distribution of Fractional Anisotropy (FA)

The percentage differences between schizophrenia and control in FA along the TBSS skeleton were calculated in each region of the White Matter Atlas (Additional file 1: Table S1).

The greatest FA reductions (>2%) were in: (i) left and right superior fronto-occipital fasciculus (SFOF)/ALIC (left -5%, p < 0.05 uncorrected; right -2.5%), (ii) left ALIC (-2.4%; p < 0.05 uncorrected), (iii) genu of corpus callosum (-3.1%; p < 0.05 uncorrected), (iv) left anterior corona radiata (-3.3%), (v) right posterior thalamic radiation (-3.2%; p < 0.05 uncorrected) and right tapetum (-2.2%), (vi) body of corpus callosum (-2.1%). No difference was significant after applying correction for multiple comparisons (FDR < 0.05).

**Figure 2:**
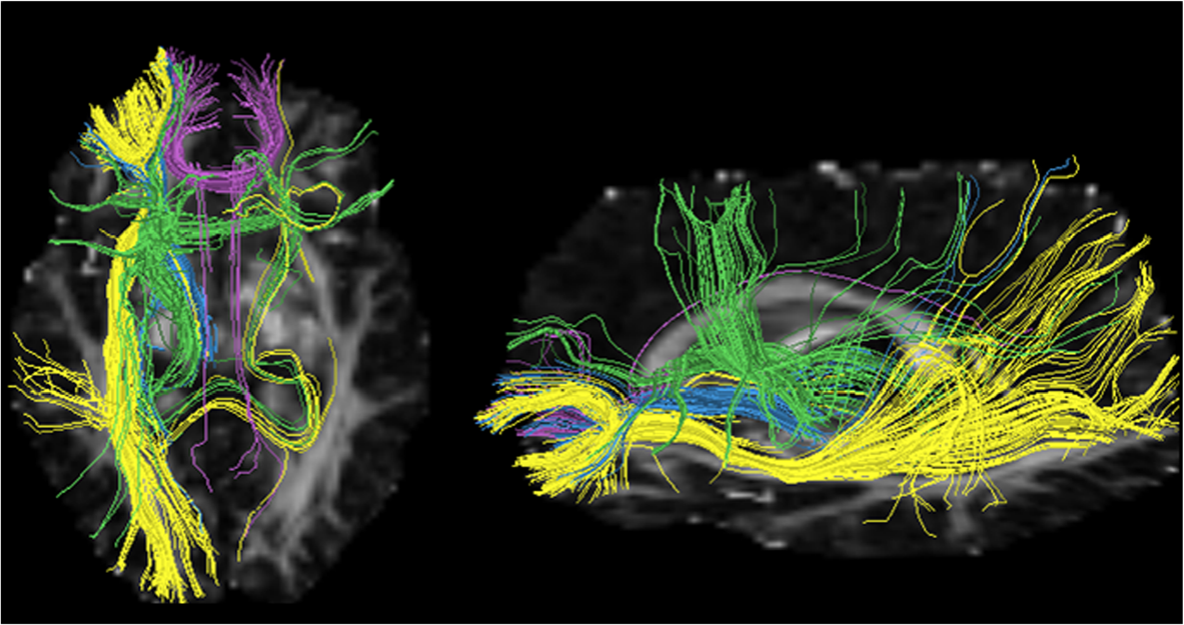
**Tracts passing through atlas regions with greatest percentage reductions of fractional anisotropy schizophrenia patients.** Tracts passing through atlas regions: genu of corpus callosum (atlas region 3; purple), left anterior corona radiata (atlas region 23, yellow) and anterior limb of the internal capsule (atlas region 17, blue), left superior fronto-occipital fasciculus/anterior limb of the internal capsule (atlas region 43, green). Tract segments displayed using DTIQuery software [25].

**Figure 3:**
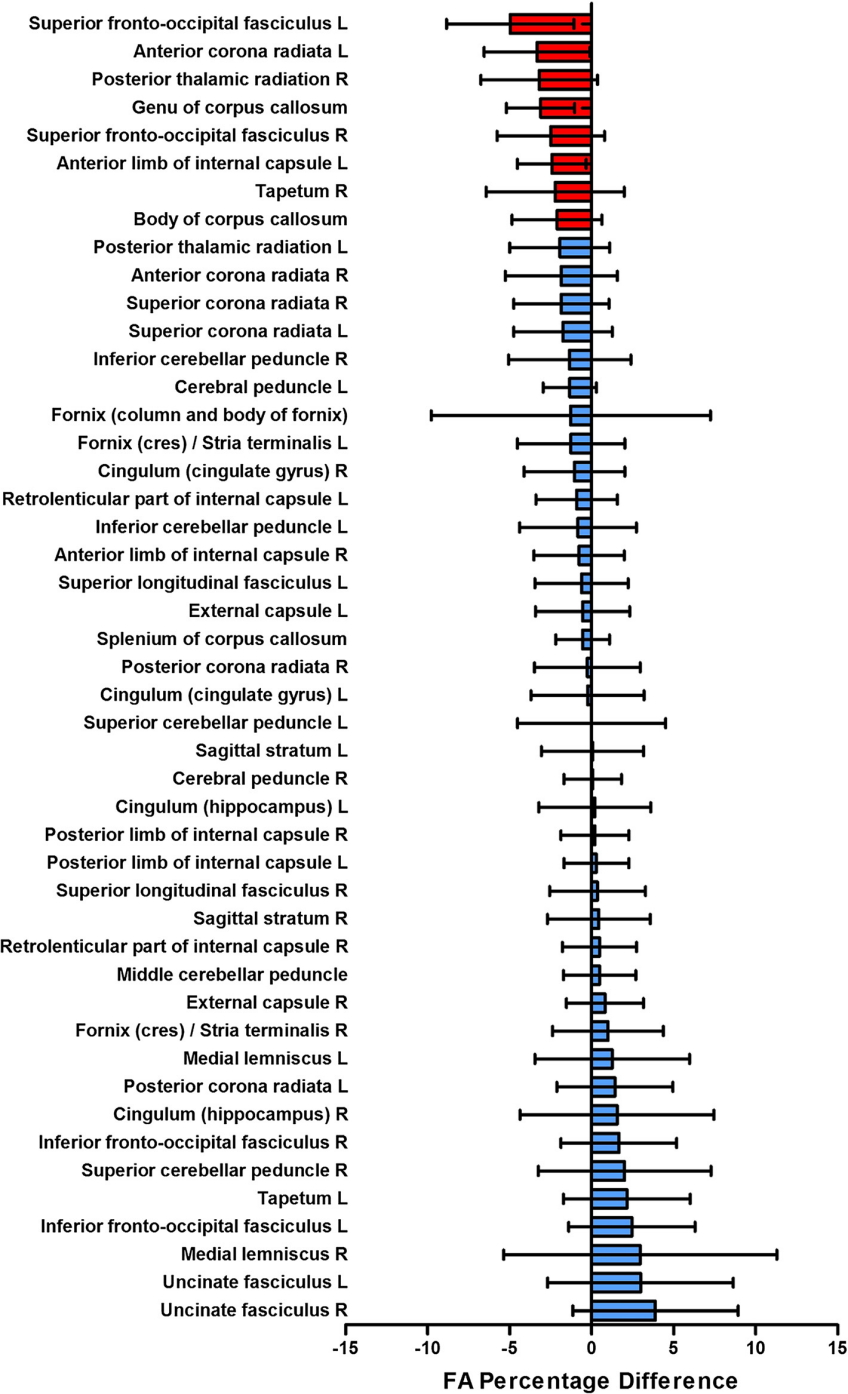
**Mean percentage differences in fractional anisotropy between schizophrenia patients and controls in standard atlas regions.** Coloured bars show mean percentage difference in fractional anisotropy between patients and controls in each standard atlas region intersecting with the Tract-based spatial statistics skeleton (regions with differences greater than 2% coloured red, others blue). Error bars depict the 95% confidence values across each atlas region. The percent is negative when the fractional anisotropy is lower in patients than controls and positive when it is larger in patients than controls.

The locations and tracts corresponding to the genu of corpus callosum (atlas region 3), left anterior corona radiata (atlas region 23), left ALIC (atlas region 17) and left SFOF/ALIC (atlas region 43) are illustrated in Figures 1 and 2. The results for all regions are shown in Figure 3.

### Tractography-based analysis of Fractional Anisotropy (FA)

Median FA for each tract examined was normally distributed (W = 0.97-0.98, p = 0.26-0.61).

FA was significantly lower in the schizophrenia (mean 0.68, SD 0.035) relative to the control group (mean 0.71, SD 0.027) in the genu of the corpus callosum (ANCOVA F(1) = 10.025, p = 0.003). FA was slightly reduced in the schizophrenia relative to the control group in the left ALIC (schizophrenia mean 0.55, SD 0.038, control mean 0.56, SD 0.035, ANCOVA F(1) = 0.048, p = 0.83) and right ALIC (schizophrenia mean 0.53, SD 0.045, control mean 0.55, SD 0.036, ANCOVA F(1) = 0.40, p = 0.53). These reductions were not significant. There were no differences between groups in the splenium (schizophrenia mean 0.73, SD 0.045, control mean 0.73, SD 0.035, ANCOVA F(1) = 0.003, p = 0.96) or the cingulum bundle (left: schizophrenia mean 0.58, SD 0.044, control mean 0.57, SD 0.036, ANCOVA F(1) = 0.323, p = 0.57; right: schizophrenia mean 0.62, SD 0.045, control mean 0.61, SD 0.031, ANCOVA F(1) = 0.329, p = 0.57). The number of reconstructed tracts in the superior longitudinal fasciculus and body of the corpus callosum were too few for analysis using tensor-based tractography.

## Discussion

This study identified altered white matter tract organization in schizophrenia, with a predominance of changes in medial frontal regions, compared to controls. Consistent with our primary hypothesis, abnormal diffusion properties were present in the Anterior Limb of the Internal Capsule (ALIC). The reductions extended beyond this and were also present in the genu, body and splenium of the corpus callosum, the left superior fronto-occipital fasciculus, and the corona radiata.

The magnitudes of the white matter diffusion changes were quantified using an atlas- parcellation method. The greatest FA reductions were in the left and right superior fronto-occipital fasciculus/ALIC, left ALIC, genu and body of the corpus callosum, left anterior corona radiata, the right posterior thalamic radiation and right tapetum parcels. The individual tracts where there were local changes identified by TBSS were examined using tensor-based tractography. FA was significantly lower in schizophrenia in tracts traversing the genu of the corpus callosum (Figure 1). The region of diffusion abnormality in the left ALIC and genu of the corpus callosum identified in this study overlapped with an area of maximal FA reduction in the deep frontal lobe white matter identified by a meta-analysis of 15 previous studies [19].

### Interpretation

The white matter changes identified in this study are consistent with a model of schizophrenia involving pathological deficits in medial frontal circuits, including a prefrontal-thalamic loop (via the ALIC) and a prefrontal-prefrontal loop (via the genu of the corpus callosum).

In support of this, several studies have found reduced FA in the anterior limb of the internal capsule (ALIC) [41,46,59-63], a region which contains several tracts, including cortico-striatal and thalamo-prefrontal fibres (anterior thalamic radiation: ATR). Reduced connectivity of the thalamus to the prefrontal cortex in schizophrenia has been found using both DTI [13] and functional imaging [64].

Other studies have identified FA reductions in the corpus callosum genu [34,65-71] and forceps minor [15,72]. These fibres provide interconnections between prefrontal regions [73]. A multi-modal study examined gray matter, white matter (using DTI) and function (using fMRI during performance of a memory task). This found white matter deficits in the genu of the corpus callosum, with projections to medial frontal regions, in association with gray matter deficits and hypoactivation during fMRI [20].

Compared with previous studies of FA changes in schizophrenia, this study found changes in medial frontal regions but did not identify FA reductions in other regions which have been previously implicated. As the sample size was small, this may indicate that the medial frontal regions are the areas of maximal FA change in schizophrenia.

Another notable aspect of the results was that we did not find gray matter reductions in patients compared with controls, unlike many previous studies of gray matter changes in schizophrenia. As discussed below, this may be attributable to the high proportion of patients treated with atypical antipsychotics. It suggests that in these patients, subtle diffusion changes may be easier to detect than volumetric changes.

### Comparison of diffusion analyses

The study applied three different analyses to the diffusion methods which provided complementary perspectives in investigating white matter changes. The methods were consistent in identifying the corpus callosum genu as a region of greater diffusion changes.

VBA and atlas-parcellation analyses identified similar regions of maximum change although the parcellation method identified some bilateral changes (left and right ALIC) when the VBA findings were lateralized (left ALIC). These differences may reflect VBA determining significance according to peak FA changes whereas the atlas-parcellation method averages FA values within the study-specific skeleton in each region. Lateralised ALIC changes in VBA (left but not right) may occur if changes are actually bilateral but right-sided changes do not reach the significance level or if the magnitude of changes are greater in the left than the right-brain [74].

### White matter volumetry

The study identified white matter changes using diffusion (in fractional anisotropy) but not volumetry (using voxel-based morphometry). White matter FA and volumetric changes in schizophrenia may not necessarily be correlated [75]. Abnormal FA may reflect changes in integrity of the myelin sheath and axonal membrane but, in general, there are many factors that can modulate the FA [26]. In neurodegenerative disorders, while FA changes often correlate with atrophy they may also be found without volumetric changes depending on the methodology, region studied and underlying pathology [76]. For example, in Alzheimer’s disease, some regions of white matter FA decrease may reflect microstructural changes rather than macroscopic changes [77] and this may be a marker for future atrophy [78]. A meta-analysis comparing white matter volumetry and DTI changes in schizophrenia suggested that DTI studies appeared more sensitive to white matter abnormalities in schizophrenia [5].

### Gray matter volumetry

Although we predicted that there would be gray matter reduction in schizophrenia in regions identified by previous studies, no significant differences between the two groups were found. Previous studies which did not detect gray matter changes were generally those investigating first-episode patients [79,80] whereas this study included patients with chronic illness.

The negative gray and white matter volumetry results may have resulted from a type 2 error as seven of 42 subjects were eliminated due to T1 data revealing motion artefacts. This could also account for the apparent greater sensitivity in detecting diffusion rather than volumetry changes.

The negative results (in contrast to most previous schizophrenia studies) might also reflect atypicality in the patient sample - for example, all patients were receiving atypical antipsychotics. Antipsychotic treatment has been associated with complex regional gray matter changes (both increases and decreases) [81]; however, gray matter loss may be less intense and widespread in patients treated with atypical antipsychotics (olanzapine) compared to typical antipsychotics (haloperidol) [82]. 8 of 21 patients in our sample were prescribed clozapine and clozapine treatment has been associated both with gray matter increases [83] or with less gray matter loss over time [84]. A study of first-episode psychosis subjects treated with atypical antipsychotics also found FA decreases but no gray matter changes [80] whereas studies of medication-naïve patients with schizophrenia have found evidence of gray matter deficits [44,85]. It is also of note that the mean premorbid IQ of the patients was high; higher performance IQ has been positively correlated with FA in schizophrenia [86].

### Further research

In the future, several approaches may help construct more detailed models of the white matter tract changes in schizophrenia.

The discrimination of white matter tracts may be improved using higher field strengths for MRI. In addition, higher angular resolution diffusion MRI (HARDI) can be used to separate and map the anterior thalamic radiation and prefronto-caudo-thalamic pathways [87] and more advanced tractography methods can be applied [88]. In addition, the classification of white matter tracts is changing, for example, the identification in humans of new tracts associated with language (the middle longitudinal fasciculus, MdLF, and Extreme Capsule, EC) [89,90].

New analyses are being used to map tract changes in areas of abnormal white matter. Theoretically, as well as a general reduction in fibers within a tract, fibers may terminate in an abnormal location, for example stopping short of their usual destination, showing altered dispersion within the destination zone [68,91] or re-routing to an abnormal destination. For example, a DTI study of Williams syndrome (WS), a rare genetic disorder arising from a hemideletion on chromosome 7q11.23, identified fiber tracts following abnormal routes [92]. A study of children with histories of early deprivation found that fronto-striatal fibers showed a more diffuse cortical distribution pattern [91].

The power to detect and define white matter changes will be improved by the analysis of larger data sets. The release of anonymised image data by a number of research groups allows testing of different image analysis methods. The results of published studies can be combined either by meta-analysis of co-ordinates (e.g. using techniques such as ALE, [93], SDM, [94], or GSMA, [95]) or changes quantified within parcellated brain regions. The latter will require publication of results, as in this study, according to standard white matter atlases. The identification of a characteristic spatial pattern of white matter tract differences may assist in earlier diagnosis using automated classification algorithms applied to neuro-images [23].

Finally, white matter tract changes in schizophrenia may be relevant to pattern of structural brain changes as the disease progresses over time. Meta-analyses of longitudinal neuro-imaging studies have found evidence for spatial progression of structural brain changes in schizophrenia [96]. Recent studies of neurodegenerative disorders have suggested that the spatial pattern of disease progression is constrained by the connectivity of large-scale neural networks [97]. The ‘spread’ of diseases such as Alzheimer’s disease and fronto-temporal dementia may be determined by pathological processes interacting with the white matter network in a disease-specific manner [98]. In this model, white matter tracts determine the anatomy of disease progression, for example by direct transneural effects, rather than spatially dispersed gray matter regions undergoing progressive change due to intrinsic vulnerability (e.g. shared neuro-chemical processes). There are similarities between the spatial pattern of gray matter changes in schizophrenia and fronto-temporal dementia [99]. Although their neuropathology may be different, the pathological effects may interact with a common white matter network in the two disorders, leading to similarities in the spatial pattern of brain changes.

### Limitations

According to the model we have proposed, medial frontal circuits are specifically affected by the pathology of schizophrenia and implicate the prefrontal cortex and connected subcortical structures (thalamus and striatum) as potential sites for the primary pathology. However, other interpretations are possible.

Firstly some researchers have argued that white matter changes in schizophrenia are global (and potentially microscopic rather than macroscopic) [16]. Some studies have found multiple white matter changes (FA reductions) within the brain in schizophrenia [23,28,100]. A meta-analysis of 23 voxel-based FA studies found that the region reported as abnormal in highest number of studies (splenium of corpus callosum) was identified in only 8 of the 23 reports [16]. However, it is possible that regional differences are superimposed on global changes. Furthermore, some white matter tracts pass through multiple regions [101,102] and deficits in these tracts may lead to the appearance of widely distributed white matter changes.

Secondly, this study may be underpowered to detect all the white matter changes present. In the meta-analysis of voxel-based FA studies of schizophrenia, two regions of white matter reduction were found in schizophrenia [19]. One was in deep frontal white matter and the second was in the left temporal lobe. The second overlapped with the white matter atlas regions of the left fornix and left retrolenticular part of internal capsule (Additional file 1: Table S1). In the current study, these regions were reduced in schizophrenia (by 1.3% and 0.9%, respectively) although these changes were not significant. Therefore there may be other white matter circuits affected in schizophrenia, including those linking limbic regions such as the hippocampus (and abnormalities in limbic regions can also be associated with frontal white matter changes, as in Temporal Lobe Epilepsy, [22]). In addition, tractography was limited by the quality of diffusion data and it was not possible to include full analyses of a number of tracts which previous studies have found to be abnormal in schizophrenia.

Thirdly, if white matter changes are present, they may not represent the specific pathology of schizophrenia but instead a consequential phenomenon. There is increasing evidence for the plasticity of brain structure in response to psychological processes and medication. For example, studies have identified gray matter changes in subjects with depression [103]. Therefore some structural changes in schizophrenia could result from the symptoms of the illness rather than represent primary pathology. For example, if increased psychotic symptoms cause FA decreases, then there may be a correlation between symptomatology and brain structural measures. We conducted an exploratory test on our data by testing for a correlation between corpus callosum genu median FA and PANSS score (a measure of psychotic symptoms). We found a weak negative correlation which was not significant (Pearson’s bivariate correlation -0.26, p = 0.08). These effects may be further elucidated by examining potential confounders or rescanning subjects during periods of exacerbation and remission of symptoms.

There were a number of other potential methodological limitations. The use of antipsychotic treatment in the patients may be a confounding factor so diffusion changes could potentially be due to the effect of medication rather than diagnosis. FA decreases have been found in the anterior cingulate and right corona radiata in previously drug-naive patients with schizophrenia after six weeks of treatment [104]. Other potential confounders are age [105-107], IQ [108], gender [109], and handedness [110]. In our data, examining the corpus callosum genu median FA as a measure of structural change, age was negatively correlated, (Pearson’s bivariate correlation -0.38, p = 0.01, whole group) and IQ was positively correlated (Pearson’s bivariate correlation 0.31, p = 0.05, whole group) as expected. The patient and control groups in our study were well matched for age and IQ so this should not have affected our results. In this study, corpus callosum genu median FA did not differ significantly by gender (*t*-test t(41)-0.86, p = 0.39) – the average FA was 2% greater in males than females (whole group). The slightly greater proportion of males in the schizophrenia group would be expected to result in higher FA values [109], rather than reduced FA values (which we detected) so this slight gender imbalance may have reduced our power to detect changes.

## Conclusions

The study located white matter tract deficits in medial frontal regions (left anterior limb of the internal capsule, genu of the corpus callosum, frontal lobe portions of the left superior fronto-occipital fasciculus and left anterior corona radiata) and the body and splenium of the corpus callosum. Tractography found deficits in the genu of the corpus callosum.

The results were consistent with a model of pathological deficits in schizophrenia in medial frontal circuits, including a prefrontal-thalamic loop (via the anterior limb of the internal capsule) and a prefrontal-prefrontal loop (via the genu of the corpus callosum).

However, further studies will be required to replicate these results and to explore the significance of white matter changes elsewhere in the brain.

## Competing interests

ETB and CD are employees of GSK and hold shares in the company. PJN was an employee of GSK at the time of the study and holds shares in the company. PJN is an employee of UCB Pharma S.A.

## Authors’ contributions

IEW, PJN, ETB, DMC designed the study. RZ, RD, MA, EF-E, CMD, UM, acquired the data which NJF, CS, AL, CM and DMC analyzed. IEW, PJN, ETB, DMC and AL wrote the article, which RZ, RD, MA, EF-E, CMD, UM, NJF, CS and CD reviewed. All authors approved its publication.

## Pre-publication history

The pre-publication history for this paper can be accessed here:

http://www.biomedcentral.com/1471-244X/14/99/prepub

## Supplementary Material

Additional file 1: Table S1 Fractional anisotropy in the schizophrenia group and controls within standard white matter atlas regions. Click here for file
