# Supplementary material for: Distribution of tract deficits in schizophrenia
Source: BMC Psychiatry. 2014 Apr 2;14:99. doi: 10.1186/1471-244X-14-99 (PMC4108049; doi:10.1186/1471-244X-14-99)
Supplement: Additional file 1: Table S1 — Fractional anisotropy in the schizophrenia group and controls within standard white matter atlas regions. [file 1471-244X-14-99-S1.doc]

**Additional file**

**Table S1.** Fractional anisotropy in the schizophrenia group and controls within standard white matter atlas regions.

| Atlas  reference | Region | Mean control | Std control | Mean patient | Std patient | %diff | 95% CI % diff | p<0.05 uncorr |
| --- | --- | --- | --- | --- | --- | --- | --- | --- |
| **43** | **SFOF-L: superior fronto-occipital fasciculus/ALIC** | **0.53** | **0.03** | **0.50** | **0.04** | **-5.0** | **3.9** | **0.01** |
| **23** | **ACR-L: anterior corona radiate** | **0.53** | **0.02** | **0.51** | **0.03** | **-3.3** | **3.2** | **0.04** |
| **30** | **PTR-R: posterior thalamic radiation** | **0.64** | **0.03** | **0.62** | **0.05** | **-3.2** | **3.6** | **-** |
| **3** | **GCC: genu of corpus callosum** | **0.75** | **0.02** | **0.73** | **0.03** | **-3.1** | **2.1** | **0.00** |
| **44** | **SFOF-R: superior fronto-occipital fasciculus/ALIC** | **0.51** | **0.03** | **0.49** | **0.03** | **-2.5** | **3.3** | **-** |
| **17** | **ALIC: anterior limb of the internal capsule L** | **0.64** | **0.02** | **0.63** | **0.03** | **-2.4** | **2.1** | **0.03** |
| **50** | **tapetum R** | **0.82** | **0.04** | **0.80** | **0.07** | **-2.2** | **4.2** | **-** |
| **4** | **BCC: body of corpus callosum** | **0.71** | **0.03** | **0.70** | **0.04** | **-2.1** | **2.7** | **-** |
| 29 | posterior thalamic radiation L | 0.63 | 0.03 | 0.61 | 0.03 | -1.9 | 3.1 | - |
| 24 | anterior corona radiata R | 0.55 | 0.03 | 0.54 | 0.03 | -1.8 | 3.4 | - |
| 26 | superior corona radiata R | 0.53 | 0.02 | 0.52 | 0.03 | -1.8 | 2.9 | - |
| 25 | superior corona radiata L | 0.52 | 0.02 | 0.52 | 0.03 | -1.7 | 3.0 | - |
| 12 | inferior cerebellar peduncle R | 0.55 | 0.03 | 0.55 | 0.03 | -1.3 | 3.7 |  |
| 15 | cerebral peduncle L | 0.74 | 0.02 | 0.73 | 0.02 | -1.3 | 1.6 | - |
| 6 | fornix (column and body of fornix) | 0.52 | 0.07 | 0.52 | 0.07 | -1.3 | 8.5 | - |
| 39 | fornix (cres) / Stria terminalis L | 0.59 | 0.02 | 0.59 | 0.04 | -1.3 | 3.3 | - |
| 36 | cingulum (cingulate gyrus) R | 0.67 | 0.03 | 0.66 | 0.04 | -1.0 | 3.1 | - |
| 21 | retrolenticular part of internal capsule L | 0.65 | 0.02 | 0.64 | 0.03 | -0.9 | 2.5 | - |
| 11 | inferior cerebellar peduncle L | 0.54 | 0.03 | 0.54 | 0.03 | -0.8 | 3.6 | - |
| 18 | anterior limb of the internal capsule R | 0.61 | 0.02 | 0.61 | 0.03 | -0.8 | 2.7 | - |
| 41 | superior longitudinal fasciculus L | 0.56 | 0.02 | 0.55 | 0.03 | -0.6 | 2.8 | - |
| 33 | external capsule L | 0.45 | 0.02 | 0.45 | 0.02 | -0.5 | 2.9 | - |
| 5 | splenium of corpus callosum | 0.78 | 0.02 | 0.77 | 0.02 | -0.5 | 1.6 | - |
| 28 | posterior corona radiata R | 0.52 | 0.03 | 0.52 | 0.03 | -0.3 | 3.2 | - |
| 35 | cingulum (cingulate gyrus) L | 0.61 | 0.03 | 0.61 | 0.04 | -0.2 | 3.4 | - |
| 13 | superior cerebellar peduncle L | 0.57 | 0.04 | 0.57 | 0.04 | 0.0 | 4.5 | - |
| 31 | sagittal stratum (includes IFL and IFOF) L | 0.58 | 0.03 | 0.58 | 0.03 | 0.1 | 3.1 | - |
| 16 | cerebral peduncle R | 0.70 | 0.02 | 0.70 | 0.02 | 0.1 | 1.7 | - |
| 37 | cingulum (hippocampus) L | 0.61 | 0.04 | 0.62 | 0.03 | 0.2 | 3.4 | - |
| 20 | posterior limb of the internal capsule R | 0.68 | 0.02 | 0.68 | 0.02 | 0.2 | 2.1 | - |
| 19 | posterior limb of the internal capsule L | 0.71 | 0.02 | 0.71 | 0.02 | 0.3 | 2.0 | - |
| 42 | superior longitudinal fasciculus R | 0.54 | 0.02 | 0.54 | 0.03 | 0.4 | 2.9 | - |
| 32 | sagittal stratum (includes IFL and IFOF) R | 0.59 | 0.03 | 0.59 | 0.03 | 0.4 | 3.1 | - |
| 22 | retrolenticular part of internal capsule R | 0.63 | 0.02 | 0.63 | 0.03 | 0.5 | 2.3 | - |
| 1 | middle cerebellar peduncle | 0.55 | 0.02 | 0.56 | 0.02 | 0.5 | 2.2 | - |
| 34 | external capsule R | 0.48 | 0.01 | 0.48 | 0.02 | 0.8 | 2.3 | - |
| 40 | fornix (cres) / Stria terminalis R | 0.60 | 0.03 | 0.61 | 0.04 | 1.0 | 3.4 | - |
| 9 | medial lemniscus L | 0.56 | 0.04 | 0.56 | 0.05 | 1.3 | 4.7 | - |
| 27 | posterior corona radiata L | 0.52 | 0.03 | 0.53 | 0.03 | 1.4 | 3.5 | - |
| 38 | cingulum (hippocampus) R | 0.58 | 0.05 | 0.59 | 0.06 | 1.6 | 5.9 | - |
| 46 | Inferior fronto-occipital fasciculus right | 0.54 | 0.02 | 0.55 | 0.04 | 1.7 | 3.5 | - |
| 14 | superior cerebellar peduncle R | 0.56 | 0.05 | 0.57 | 0.04 | 2.0 | 5.3 | - |
| 49 | tapetum L | 0.61 | 0.04 | 0.62 | 0.03 | 2.2 | 3.9 | - |
| 45 | Inferior fronto-occipital fasciculus left | 0.54 | 0.02 | 0.56 | 0.04 | 2.5 | 3.8 | - |
| 10 | medial lemniscus R | 0.51 | 0.08 | 0.53 | 0.06 | 3.0 | 8.3 | - |
| 47 | uncinate fasciculus L | 0.53 | 0.04 | 0.55 | 0.05 | 3.0 | 5.7 | - |
| 48 | uncinate fasciculus R | 0.46 | 0.03 | 0.48 | 0.05 | 3.9 | 5.0 | - |
| 2 | Pontine crossing tract (a part of MCP) | - | - | - | - | - | - | - |
| 7 | Corticospinal tract left | - | - | - | - | - | - | - |
| 8 | Corticospinal tract right | - | - | - | - | - | - | - |

Percentage reductions in FA in the schizophrenia group are shown for each parcel (regions where % difference greater than 2% shown in bold). Abbreviations: R (Right), L (left), IFL (Inferior Longitudinal Fasciculus), IFOF (Inferior Fronto-Occipital Fasciculus), SFOF (Superior Fronto-Occipital Fasciculus), MCP (Middle Cerebellar Peduncle). The three regions at the end did not intersect with the Tract-based spatial statistics map so no data are given.
